# 

**Published:** 1920-08-07

**Authors:** 


### The Soldiers Still in Hospital.

In co-operation with the Ministry of Pensions, the Y.M.C.A. is doing a useful piece of work at the Ducane Road Hospital, Wood Lane, W., where from 800 to 1,200 men are awaiting or undergoing surgical treatment, operations, and amputations. One of the sights of the week is to see the bringing down of the bed-patients one by one to the Y.M.C.A. Hut, where they all enjoy the concerts, chess tournaments, and various competitions for their entertainment. The hut is equipped with four billiard tables, and is run by a lady who served the Y.M.C.A. overseas during almost the whole of the war. The authorities expect that the work at this centre will last for four years more.

### The College of Nursing.

**(Limited by Guarantee.)**

### YORKSHIRE CENTRE.

Garden Party at Chapel Allerton.

About fifty members spent an enjoyable afternoon at Stainbeck Hospital, Chapel Allerton, Leeds, on Saturday, July 24, when Miss Innes and Miss Bayley entertained the members to a garden party. The weather was beautiful and sunny, and the grounds presented a fresh and charming appearance. Tennis, croquet, and clock golf were provided for those wishing to play, and there were many circles of friends sitting about enjoying the sun, which has been all too rare lately. Tea was served in a room overlooking the gardens, and later ices were much appreciated. After tea the room was cleared and some of the members danced; others played tennis or strolled about the grounds.

Arrangements for Picnic, August 19.

A picnic to Bolton Woods by motor char-à-banc on Thursday, August 19, has been arranged, members paying 5s., non-members 11s., including tea. The motor char-à-banc will leave Great George Street, Leeds, by the Leeds General Infirmary and St. George's Church, at 12 noon punctually. Members should either lunch before starting or take it with them. They are requested to send in their names to the Hon. Sec., Miss Lindall, Hospital for Women and Children, Leeds, not later than Thursday, August 12. Membership cards must be produced.

### NORTHUMBERLAND AND DURHAM CENTRE.

The Northumberland and Durham Nurses' Residential Club at Newcastle-on-Tyne, under the auspices of the College of Nursing Centre for the two counties, was thrown open to members on Wednesday, July 28. The new Club, which is at 17 Windsor Terrace, in one of the prettiest parts of Jesmond, is a large house. The commodious rooms have been admirably adapted, and simply, but very comfortably, appointed and furnished. The cosy lounge contains a good supply of magazines, papers, and books, and there is good bedroom accommodation. Refreshments have been arranged for, and light meals will be obtainable in the dining-room at fixed hours. The Club will be open from nine o'clock in the morning until ten at night, and membership is open to certificated nurses, trained in general, fever, or children's work. Nurses in their fourth year of training in a recognised school may also become members of the Club, which promises already to be a very popular rendezvous for the profession.

### IRISH BOARD.

The office of the Irish Board of the College, 54 Fitzwilliam Square, Dublin, will be closed during August.

### Matrons' and Sisters' Appointments.

Ashurst War Hospital, Littlemore, near Oxford.—Miss E. C. Hayward has been appointed sister. She was trained at the General Hospital, Walsall, and served in the Queen Alexandra's Royal Military Nursing Service Reserve, and was sister at the Military Hospital, Grosvenor Road, London. Miss L. Davies Maddox has been appointed sister. She was trained at Hahnemann Hospital, Liverpool, and has since been sister at Monsall Hospital, Manchester. She also served at the 1st Eastern General Hospital, Cambridge, and at the 21st General Hospital, Alexandria.

City Fever Hospital, Coventry.—Miss Gladys M. Batteson has been appointed night sister. She was trained at the Royal Infirmary, Doncaster, and in fever work at Doncaster and Mexborough Joint Hospital. She has since been staff nurse at Walsall General Hospital. She is a member of the College of Nursing.

East Suffolk and Ipswich Hospital, Ipswich.—Miss E. Funnell has been appointed sister. She was trained at the East Suffolk and Ipswich Hospital, and has since been staff nurse at the Cottage Hospital, Felixtowe; staff nurse and, later, sister at the War Hospital, Gosforth; temporary night sister at Kirkcaldy Hospital, Fifeshire; and staff nurse at Lewisham Hospital.

Maternity Hospital, Swansea.—Miss Florence M. Julian has been appointed sister. She was trained at Cardiff Union Hospital, and has since been staff nurse at Mountain Ash Hospital; nurse-in-charge, Taunton Union; sister, Queen Alexandra's Imperial Military Nursing Service Reserve; district midwife, Tredegar; and sister at Bellshill Maternity and Child Welfare Hospital.

West Cornwall Infirmary, Penzance.—Miss Amy M. Bignell, A.R.R.C., has been appointed sister. She was trained at the Dreadnought Hospital, Greenwich, and Hospital for Women, Soho Square, London, and in fever work at Monsall Fever Hospital, Manchester. She has since been sister, Territorial Force Nursing Service, at King's College Hospital, London, and night superintendent at North Devon Hospital, Barnstaple. She is a member of the College of Nursing.

### Salaries of Health Visitors.

The Women Sanitary Inspectors' and Health Visitors' Association, 1 Upper Montague Street, W.C. 1, forwards us the following for publication:

The above Association desires to draw the attention of intending applicants for posts under public-health authorities to the following facts: (1) That in August 1918 the Local Government Board, in their maternal and child-welfare circular, advised that health visitors should be paid a salary of not less than £120 per annum. (2) Since then there have been several Civil Service awards, which the Government have recommended local authorities to adopt. (3) That on May 8 the Cost of Living Committee of the National Whitley Council recommended the following bonus to supersede all previous ones: (*a*) Where the ordinary rate of pay does not exceed £91 5s. per annum, 130 per cent. of ordinary remuneration. (*b*) Where the ordinary rate of remuneration exceeds £91 5s., but does not exceed £200 per annum, 130 per cent. on the first £91 5s., and 60 per cent. on such amount as is in excess of £91 5s. (4) This bonus brings a salary of £120 per annum up to £255 19s. 6d., which, in the opinion of the National Whitley Council, would justly meet the increased cost of living. (5) In view of these facts the Vigilance Committee of this Association warns intending applicants against applying for posts where inadequate salaries are offered. In several instances where no applications were received, other advertisements had to be inserted offering an improved salary.

### Hospital for Consumption and Diseases of the Chest,

BROMPTON, S.W. 3 (NEAR SOUTH KENSINGTON STATION).

### LECTURES ON TUBERCULOSIS.

AUTUMN SESSION.—TUESDAYS and FRIDAYS at 8 p.m.

October 12 (1) "Anatomy and Physiology of the Chest," A. Hope Gosse, M.A., M.D., M.R.C.P. Oct. 15 (2) "Distribution and Incidence of Tuberculosis," L. S. T. Burrell, M.A., M.D., M.R.C.P. Oct. 19 (3) "Immunity and Infection," A. L. Punch, M.B., B.S., M.R.C.P. Oct. 22 (4) "Causes of Tuberculosis," W. B. Knobel, M.A., M.D., M.R.C.P. Oct. 26 (5) "General Signs and Symptoms of Pulmonary Tuberculosis," W. J. Fenton, M.D., F.R.C.P. Oct. 29 (6) "The *X*-Ray Evidence," Stanley Melville, M.D.

November 2 (7) "Nose and Throat in relation to Tuberculosis," Sir James Dundas-Grant, M.D., F.R.C.S. Nov. 5 (8) "Pathological Anatomy of Pulmonary Tuberculosis," A. L. Punch, M.B., B.S., M.R.C.P. Nov. 9 (9) "Principles of Treatment of Pulmonary Tuberculosis," G. E. Beaumont, M.A., M.D., M.R.C.P. Nov. 12 (10) "Nursing of Pulmonary Tuberculosis in Hospital," W. B. Knobel, M.A., M.D., M.R.C.P. Nov. 16 (11) "Tuberculosis in Children," R. C. Wingfield, M.A., M.D., M.R.C.P. Nov. 19 (12) "Surgical Tuberculosis," J. E. H. Roberts, F.R.C.S. Nov. 23 (13) "Principles of Social Work," Miss Marx. Nov. 26 (14) "Sanatorium Treatment and After-Care," R. C. Wingfield, M.A., M.D., M.R.C.P. Nov. 30 (15) "Home Nursing of Tuberculosis and Dispensary Management," Miss Redl.

December 3 (16) "Prevention of Tuberculosis," W. B. Knobel, M.A., M.D., M.R.C.P. Dec. 7 (17) "Economic Factors in Tuberculosis," Miss Marx. Dec. 10 (18) "History of Tuberculosis," L. S. T. Burrell, M.A., M.D., M.R.C.P.

Fee for Course, £1 1s. Single Lecture, 2s. Fee for Course with Demonstrations and Examination, £2 2s.

Demonstrations.—In Laboratory, Bacteria and Staining; in Museum, Pathological Specimens; in Wards, Nursing; in Dispensary and Out-Patient Departments.

